# A comparative study of cyclic fatigue of 6 endodontic systems. An *in vitro* study

**DOI:** 10.4317/jced.59747

**Published:** 2022-07-01

**Authors:** Jorge Rubio, José-Ignacio Zarzosa, Susana Aranda, Alberto Casino, Antonio Pallarés

**Affiliations:** 1 Professors of the Master of Endodontics and Restorative Dentistry, Catholic University of Valencia; 2 Director of the Master of Endodontics and Restorative Dentistry, Catholic University of Valencia

## Abstract

**Background:**

Mechanical preparation and the formation of space for adequate obturation are included in root canal shaping, but the complex root canal anatomy may be affect it. Manufacturers have created different alloys like M-Wire, Blue-Wire, Gold-Wire or R-phase. Objective: This investigation was performed to verify the null hypothesis that there were not significant differences between size 25 instruments ESP Files Thermoflex, Protaper Ultimate, Protaper Next, Blueshaper, One Curve and 2Shape about cyclic fatigue and length of broken fragments.

**Material and Methods:**

180 new size 25 files of the systems investigated were selected (n=30). Files were used with Endo Mate DT endo motor with speed and torque recommended by manufacturers, holding the instruments with clamping mechanism, with passive adjustment, glycerine and without pressure in a stainless-steel block. The time was calculated in seconds until fracture. Number of fatigue cycles was determined as (Resistance (s) x Speed)/60. Separated fragment lengths were calculated with digital Vernier caliper. Statistical analysis was carried out with the SPSS 18 programme at a 95% confidence level, using Levene´s Test to compare variances, Welch’s Test to compare means, and Games-Howell´s Test to reveal differences between groups.

**Results:**

Levene’s Test determined no equal variances (*P*<0.05). Welch’s Test and ANOVA (*P*<0.05) showed significant differences in cyclic fatigue and separated fragment lengths. Games-Howell’s and Bonferroni´s Test established significant differences in multiple comparisons (*P*<0.05).

**Conclusions:**

ESP Files Thermoflex was superior in cyclic fatigue. About separated fragment lengths, ESP Files Thermoflex, Protaper Ultimate and Blueshaper obtained longer lengths.

** Key words:**Cyclic fatigue, continuous movement, separated fragment lengths, rotary systems.

## Introduction

Mechanical preparation and the formation of space for adequate obturation are included in root canal shaping, but the complex root canal anatomy may be affect it. For this reason, different instruments and alloys were designed (1). The fracture of instruments remains a problem in root canal treatment, despite improved file performance (2). Thanks to the physical transformation of martensitic and austenitic phases, the instruments have great flexibility, but bending fatigue or torsional failure lead to fracture (3).

Manufacturers have created different alloys like M-Wire, Blue-Wire, Gold-Wire or R-phase. M-Wire increases resistance to fracture of conventional NiTi with heat treatment, but files have elastic memory (4). However, instruments with Gold-Wire have control memory and more resistance and flexibility than M-Wire files (5,6). The Blue-Wire of new Blueshaper files, was designed with special thermal process and stable martensitic phase, increasing the flexibility and resistance to fracture by cyclic fatigue, and avoiding elastic memory (7-9). About R-phase Thermoflex Control with heat treatment of new ESP Files system, provides to instruments with memory control and more flexibility, avoiding traction to recuperate the anatomy inside the root canal.

Apart from alloy, there are multiple factors that affect the resistance to fracture of the instruments, such as the number of uses, the apical pressure of the operator, the design or the type of movement (10,11). Regarding the length of separated fragments, it is desirable that the longest fragment remain inside the root canal to perform its removal with extractor system.

Therefore, this investigation was performed to verify the null hypothesis that there were not significant differences between size 25 instruments ESP Files Thermoflex (Jata Endo, Madrid, Spain), Protaper Ultimate (Dentsply Sirona, Ballaigues, Switzerland), Protaper Next (Dentsply Sirona, Ballaigues, Switzerland), Blueshaper (Zarc4Endo, Gijón, Spain), One Curve (Micro-Mega, Besançon, France) and 2Shape (Micro-Mega, Besançon, France) about cyclic fatigue and length of broken fragments.

## Material and Methods

Similar methods to those mentioned in another scientific paper, which published in 2019 by the same authors, were used (12).

180 new size 25 instruments of systems investigated were selected (n=30). In alphabetical order, systems were divided into groups (Table 1).

**Table 1:**
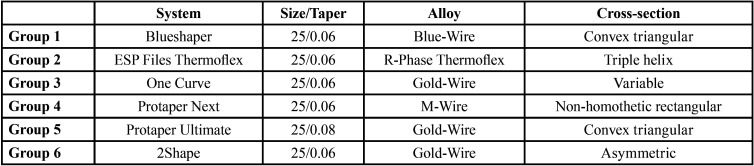
Systems and their characteristics.

**Figure 1:**
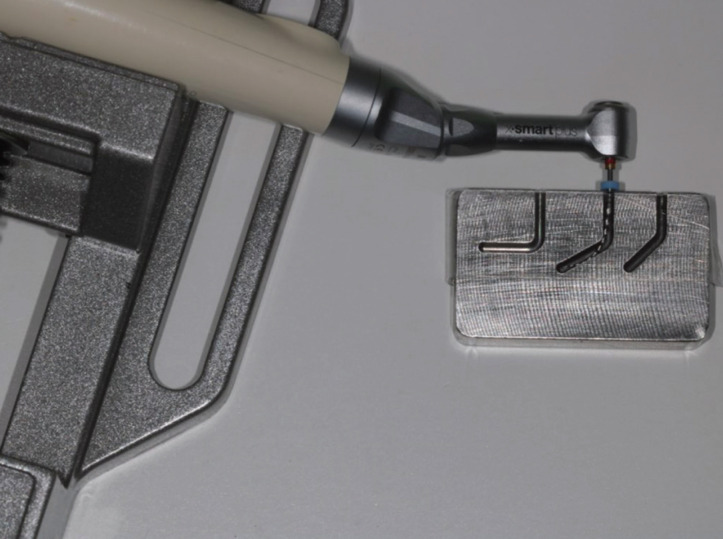
File in an artificial 60º canal.

Files were used with X-Smart Plus endo motor (Dentsply Sirona, Ballaigues, Switzerland) (Fig. 1) with speed and torque recommended by manufacturers. Speed and torque of groups with continuous rotation were:

- Group 1 (Blueshaper): 350 rpm, 4 N•cm.

- Group 2 (ESP Files Thermoflex): 300 rpm, 2 N•cm.

- Group 3 (One Curve): 400 rpm, 2,4 N•cm.

- Group 4 (Protaper Next): 300 rpm, 2 N•cm.

- Group 5 (Protaper Ultimate): 400 rpm, 4 N•cm.

- Group 6 (2Shape): 400 rpm, 2,6 N•cm.

**Figure 2:**
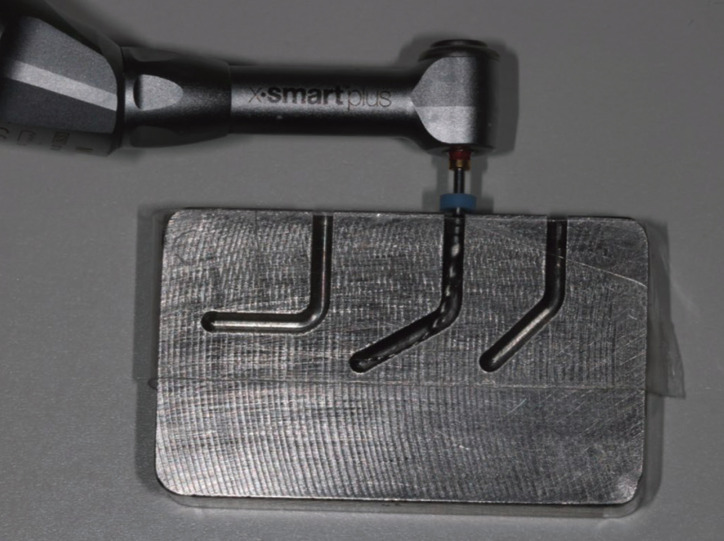
Clamping mechanism of the handpiece for the X-Smart Plus endo motor.

The instruments were firmly held with clamping mechanism (Fig. 2) without pressure and with passive adjustment in stainless-steel block with artificial canal with following characteristics: radius of curvature 3.5 mm, 60º curvature, width 2 mm, length 21 mm and depth 3 mm. Champa *et al*. (13) and Gambarini *et al*. (14) used a block with similar characteristics. The canal was lubricated with glycerine after each file.

Until fracture, time was calculated in seconds (s). With following formula, number of cycles to fracture (NCF) was estimated: (Resistance (s) x Speed)/60. With digital Vernier caliper, separated fragment lengths were calculated.

Statistical analysis was carried out with the SPSS 18 programme at a 95% confidence level, using Levene´s Test to contrast variances, Welch’s Test and ANOVA to compare means, and Games-Howell´s and Bonferroni´s Test to determine differences between groups.

## Results

No equal variances were assumed (*P*<0.05) with Levene´s Test, so Welch´s Test was used to contrast means consequently (Tables 2-4).

The cyclic fatigue mean values and statistics were described in Table 2. About statistics, ESP Files Thermoflex was superior to the other systems (*P*<0.05), but there were no significant differences between Protaper Ultimate vs Blueshaper (*P*=0.943) and 2Shape vs Blueshaper (*P*=0.270).

**Table 2:**
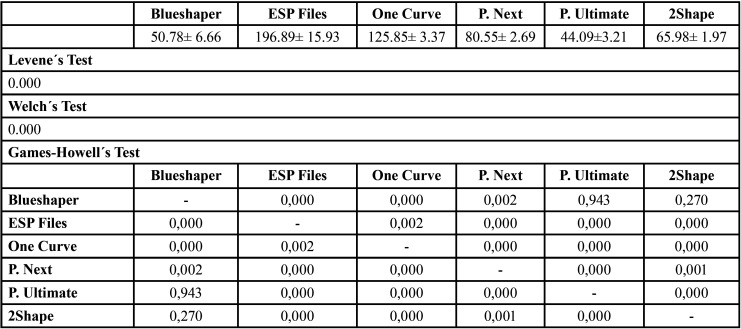
Means and statistics for resistance to cyclic fatigue (s).

The NCF were presented in Table 3. ESP Files Thermoflex was superior statistically (*P*<0.000) except in comparison with One Curve (*P*<0.506). Nevertheless, there were not significant differences between Blueshaper vs Protaper Next (*P*=0.126), 2Shape vs Protaper Next (*P*=0.373) and Protaper Ultimate vs Blueshaper (*P*=1.000).

Separated fragment lengths were described in Table 4. The largest values were acquired by ESP Files, Protaper Ultimate and Blueshaper. These systems were statistically superior (*P*=0.000) vs One Curve, Protaper Next and 2Shape.

**Table 3:**
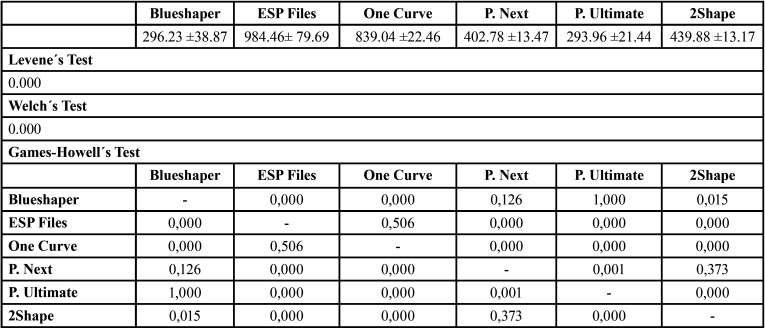
Means and statistics of number of cycles to fracture (NCF).

**Table 4:**
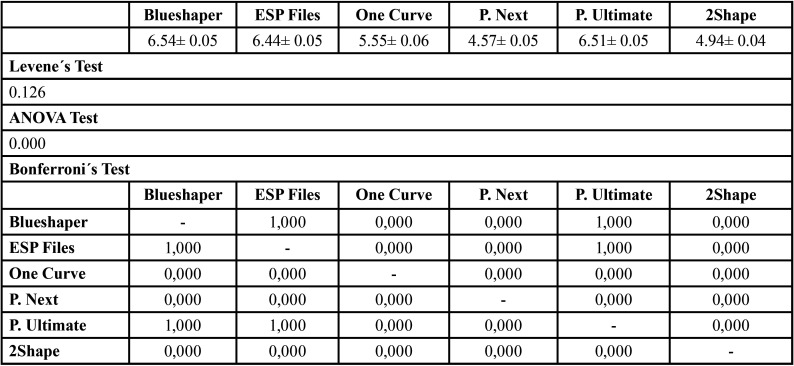
Means and statistics of separated fragments length (mm).

## Discussion

In the present research, files with continuous movement but with different cross-section, taper, alloy, torque and speed were investigated. Results showed that ESP Files with R-Phase Thermoflex obtained better resistance to cyclic fatigue.

In the 1980s, Walia *et al*. introduced NiTi instruments in endodontics, and the manufacturing process has been developing with improvements to avoid failures like broken files and to have better mechanical properties of alloy. Actually, endodontic instruments can be classified in files with higher austenite phase (NiTi, R-Phase or M-Wire), or with superior martensite phase (CM-Wire, Blue-Wire or Gold-Wire). The first files are indicated to shape slightly curved or straight canals, and the instruments with higher martensite phase are suiTable to treat severe or double curvatures of root canals, due to the greater flexibility and control memory (15-17). According to Goldberg *et al*. (18) Defects such as zipping or apical transportation can be observed in root canal treatment, with an increased risk of treatment failure.

On the other hand, metallurgical design, manufacturing process, taper, size, cross section, helix angle, file kinematics, core diameter and heat treatment of alloy are factors with which the resistance to fracture can be affected (19,20). In the present investigation, ESP Files Thermoflex (196.89±15.93s) obtained the best resistance statistically. Regarding NCF, ESP Files Thermoflex (984.46±79.69) and One Curve (839.04±22.46) showed the highest NCF.

There are several studies of cyclic fatigue with dynamic or static models. As in the investigations of Lopes *et al*. (21), Alcalde *et al*. (22), Almeida-Gomes *et al*. (23) or Wan *et al*. (24), static model was selected to reduce variables such as the amplitude of axial movements of files in dynamic model, and to have a precise position in the artificial root canal in stainless steel block.

The results of this investigation ( Table 2, Table 3) showed that Group 2 (ESP Files, 196.89±15.93s, 984.46±79.69 NCF) and 3 (One Curve, 125.85± 3.37s, 839.04±22.46 NCF) got better results than the other groups. However, Groups 1 (Blueshaper, 50.78± 6.66s, 296.23 ±38.87 NCF) and 5 (Protaper Ultimate, 44.09±3.21s, 293.96 ±21.44 NCF) obtained the worst results, which have the highest torque. About separated fragment lengths Blueshaper (6.54± 0.05mm), ESP Files (6.44± 0.05mm) and Proper Ultimate (6.51± 0.05mm) showed the highest values.

Riyahi *et al*. (25) published an investigation of cyclic fatigue comparing Protaper Next, Twisted Files and Trunatomy (Dentsply Sirona, Ballaigues, Switzerland) with same size of this study but with less instruments per group in stainless steel canal of 60º. Regarding NCF, Protaper Next (259±37.2 NCF) was less resistant statistically in comparison with Trunatomy (846.67±37.16 NCF) and Twisted Files (521.67±63.07 NCF). In other study of Chi *et al*. (26), they compared Protaper Next, Protaper Universal, Wave One Gold, Hyflex CM, Hyflec EDM and TFA Adaptive in artificial canal of 60º, and Protaper Next (61 NCF) showed the worst result also. On the other hand, Ruiz-Sánchez *et al*. (27) compared Protaper Next, Protaper Universal, Protaper Gold and Profile Vortex Blue, and they determined significant differences between groups, being Protaper Next (787.90±173.91 NCF) significant worse than Protaper Gold (1021.49±264.81 NCF) and Profile Vortex Blue (1431.46±411.60 NCF). However, in this study Protaper Next (402.78±13.47 NCF) was better statistically than Protaper Ultimate (293.96±21.44 NCF) and Blueshaper (296.23±38.87 NCF), without significant differences in comparison with 2Shape (439.88 ±13.17 NCF), and significant worse than ESP Files Thermoflex (984.46± 79.69 NCF) and One Curve (839.04 ±22.46 NCF).

Koçak *et al*. (28) performed an cyclic fatigue study of Protaper Next, 2Shape, Hyflex CM and TF Adaptive; and they observed that Protaper Next (554,7±59 NCF) was significant better than 2Shape (291±46 NCF). In 2019, Olcay *et al*. (29) published an investigation of cyclic fatigue of 2Shape, Protaper Next and Wave One Gold in simulated canal of 60º also. They determined that Protaper Next (807.0±33.43 NCF) was significant better than 2Shape (388.6±13.08 NCF) like Koçak *et al*. (28). One year after, Gündoğar *et al*. (30) studied the cyclic fatigue and separated fragment length of Rotate, Trunatomy, 2Shape and Hyflex CM files in artificial canal with similar characteristics of the block of this investigation, and they observed significant differences in cyclic fatigue between groups, being 2Shape (1155.53±173.25 NCF) less resistance than Rotate (1840.84±257.62 NCF) and Hyflex CM (1566.62±250.55 NCF), but no significant differences in separated fragment length (*P*>0.05). Unlike Koçak *et al*. (28) and Olcay *et al*. (29), the present study found no significant differences (*P*=0.373) between Protaper Next (402.78±13.47 NCF) and 2Shape (439.88 ±13.17 NCF), but significant differences in separated fragment length were observed between both systems (*P*=0.000), in contrast with Gündoğar *et al*. (30).

La Rosa *et al*. (31) examined One Curve and F6 SkyTaper endodontic files in artificial canal with a curvature of 60º also. In the results, they observed similar value of One Curve in comparison of this study (125.85±3.37 NCF). One year before, Uygun *et al*. (32) compared cyclic fatigue and separated fragment length of One Curve, Hyflex EDM, Vortex Blue and Protaper Gold. The authors observed in cyclic fatigue that One Curve (959.58±61.18 NCF) was better significantly than Vortex Blue (548.39±77.64 NCF) and Protaper Gold (600.83±66.49 NCF). About separated fragment length, they determined significant differences obtaining the shortest value the One Curve system (4.54±0.20mm). About the results of La Rosa *et al*. (31), in this investigation were showed similar results of One Curve. Regarding to Uygun *et al*. (32), One Curve was significant better than system with Gold-Wire in this study also, but One Curve (5.55±0.06mm) did not get the shortest value in separated fragment length (Protaper Next, 4.57±0.05mm).

In 2021, Sierra-Lorenzo *et al*. (33) published an investigation of cyclic fatigue comparing Blueshaper and Protaper Ultimate systems in artificial canal with curvature of 60º, like this study. In the results, they showed that Blueshaper (1205.6 NCF) was significant superior than Protaper Ultimate (736 NCF). Unlike Sierra-Lorenzo *et al*. (33), Blueshaper (296.23±38.87 NCF) and Protaper Ultimate (293.96±21.44 NCF) showed no significant differences in NCF and fracture time.

ESP Files Thermoflex was superior to other investigated systems for cyclic fatigue. About separated fragment lengths, ESP Files Thermoflex (R-Phase with heat treatment), Protaper Ultimate (Gold-Wire) and Blueshaper (Blue-Wire) obtained longer lengths.
